# Dataset of Uzbek verbs with formation and suffixes

**DOI:** 10.1016/j.dib.2025.111731

**Published:** 2025-05-30

**Authors:** Maksud Sharipov, Jernej Vičič

**Affiliations:** a Urgench State University named after Abu Rayhan Biruni, 14, Kh. Alimdjan str, Urgench city 220100, Uzbekistan; b University of Primorska, FAMNIT, Glagoljaska 8, 6000 Koper, Slovenia

**Keywords:** Verb phrase, Uzbek language, Uzbek web corpus, Verb form, Verb affixes

## Abstract

The main goal of this work is to create a dataset of Uzbek language verbs. This dataset stores information about which words verbs are derived from and with which affixes. The affixes are classified into distinct categories. With the help of this dataset, it is possible to determine from which parts of speech each Uzbek verb is derived and with which affixes. It also plays a key role in identifying verbs in Uzbek language texts and developing rule-based models for their analysis. Additionally, this dataset plays a key role in building various artificial intelligence models for the morphological and syntactic analysis of Uzbek language texts. Verbs play a crucial role in learning any language; therefore, students in schools and higher education institutions can also use this dataset during the learning process. The obtained dataset serves as a valuable resource for researchers and practitioners interested in Uzbek language processing tasks.

Specifications Table

**Table:** 

Subject	Computer Science Applications
Specific subject area	Natural language processing
Type of data	Table
Data collection	In the process of data collection, the *Explanatory Dictionary of the Uzbek Language* (5-volume edition) [1] was utilized. Information regarding affixes involved in verb formation was sourced from Uzbek language school textbooks. Experts extracted verbs and their corresponding affixes from the dictionary, systematically categorizing them into groups and compiling them into a structured table. The order of verbs, their preceding word classes, and the sequence of affixes were determined and arranged accordingly in table columns. As a result, a total of 8,502 verbs and their affixes were collected and organized.
Data source location	Higher educational institution: Urgench State University named after Abu Rayhan Biruni 14, Kh.Alimdjan str, Urgench city, 220100, Uzbekistan
Data accessibility	Repository name: Mendeley data Data identification number: 10.17632/bhmwhh95j3.1 Direct URL to data: 10.17632/bhmwhh95j3.1
Related research article	*None*

## 1 Value of the Data


 

• The dataset contains more than 8K Uzbek verbs manually annotated with their derivational and voice-related affixes (collaborative, intensive, passive, reflexive), providing a valuable linguistic resource for analyzing the structure and formation of verbs in the language, which is an essential resource for low-resource scenarios;
• Researchers in Uzbek linguistics (UL), natural language processing (NLP), as well as computational linguistics (CL) can reuse this dataset to build or enhance morphological analyzers, part-of-speech taggers, or rule-based verb identification systems built for the Uzbek language;
• In the structured format that is common for both researchers, students, and industry, with clearly labeled columns (verb base form, source part of speech, affix types), allows for easy integration into machine learning and deep learning pipelines and computational linguistics tools;
• This dataset can also become handy in educational settings to teach verb morphology, derivation processes, and affixation in the Uzbek language, which is beneficial for both linguists and language learners.

## 2 Background

The dataset of **Uzbek verbs with different forms and suffixes** presents significant value both for linguistic research and the development of Natural Language Processing (NLP) applications. Uzbek, being a Turkic language with agglutinative characteristics, showcases a remarkable variety of verb forms that play a central role in sentence construction and meaning. These verb forms, influenced by a multitude of affixes, allow the language to convey nuances in action, aspect, mood, and voice, making them essential for fully understanding the grammatical and syntactical structure of Uzbek.

In Uzbek, verbs are essential to the language's structure. The richness and diversity of the verb forms reflect the complexity of the language itself, as well as the contextual difference it expresses. In Uzbek, verbs are not limited to simple action words; they embody a wide array of meanings through their various conjugations, derivations, and transformations using prefixes and suffixes. Each verb can take on a different meaning depending on the form or suffix attached to it, showcasing the lively nature of the Uzbek language.

For example, verbs in Uzbek can change their meaning by adding **derivational affixes**, which turn a basic verb into another word class (e.g., transforming an adjective into a verb), or by using **collaborative** or **intensive voice affixes**, which alter the subject-object relationships and intensity of the action. This array of affixes enables speakers to express complex concepts with a single verb. The dataset, therefore, serves as a crucial resource to capture the full potential of Uzbek verbs and their many forms.Each of these affix types plays a unique role in shaping the meaning of the verb:

1. **Derivational Affixes**: These affixes allow the transformation of a verb into other parts of speech, such as adjectives or nouns. For example, the affix "-lash" in "abadiylashmoq" (to eternalize) transforms an adjective ("abadiy") into a verb.
2. **Voice Affixes**: Uzbek verbs utilize a variety of voice markers to alter the syntactic role of the subject. **Collaborative voice affixes** (e.g., "-moq") suggest actions that are performed in cooperation with others, while **passive voice affixes** (e.g., "-il") shift the focus from the subject performing the action to the subject receiving it. **Reflexive voice affixes** (e.g., "-lanmoq") indicate that the action is performed on the subject itself.
3. **Intensive Voice**: The addition of intensive markers (e.g., "-lashmoq") intensifies the verb's meaning, emphasizing the force or extent of the action.

The dataset is structured in a simple and accessible format (MS Excel), stored in on Mendeley data, which makes it easy to analyse, manipulate, and integrate into computational tools. The dataset includes the following key columns:

• **Verb name**: The base verb forms in their root state.
• **Previous Part of Speech**: The original part of speech from which the verb is derived (e.g., adjective, noun).
• **Derivational Affix**: The affix used to derive the verb.
• **Collaborative Voice Affixes, Intensive Voice, Passive Voice, Reflexive Voice**: These columns contain the relevant affixes attached to the verb to indicate voice and intensity.

In NLP, verbs are central to syntactic and semantic analysis, making them essential for various tasks such as machine translation, text generation, and semantic parsing. Verbs carry much of the information about the relationships between subjects and objects in a sentence. Given the agglutinative nature of Uzbek, where a single verb form can carry a lot of grammatical information through its affixes, correctly identifying and processing verbs is vital for accurate language modeling [2].

Mengliev et al. present a dataset for named entity recognition (NER) in Uzbek, consisting of 1160 sentences and 19,000 annotated word forms. The authors also develop two NER algorithms for Uzbek, contributing to NLP research and machine learning in low-resource languages. This work provides a foundation for NER in Uzbek and similar languages, supporting applications such as chatbots and text mining tools. Stop word [3] filtering is essential for efficient text processing, reducing query complexity without losing semantic content. However, existing models are often ineffective for agglutinative languages like Uzbek, where stop words are obscured by suffixes. This study introduces a collocation method for stop word identification in such languages, applying unigram, bigram, and collocation analysis to the Uzbek "School corpus" to enhance stop word filtering in agglutinative languages. An annotated and curated dataset of sentiment analysis collected from local restaurant reviews is presented in [4]. As part of the crucial datasets needed to build machine learning and deep learning models, this dataset consists of annotated restaurant reviews for Uzbek texts, which is to our knowledge, the first of its kind.

This paper [5] discusses the construction of a lemmatization algorithm for the Uzbek language. In this paper [6], a rule-based approach for detecting verbs in Uzbek texts, based on affixes and suffixes, is proposed. Maksud et al. [7] present a rule-based stemming algorithm for the Uzbek language. Sharipov et al. [8] l present a part-of-speech (POS) annotated dataset and tagger tool for the low-resource Uzbek language. The study conducted by Mercanoglu et al. [9] introduces AUTSL, a large-scale, multi-modal dataset for Turkish Sign Language (TSL) consisting of 226 signs performed by 43 signers, with 38,336 video samples captured using Microsoft Kinect v2 (RGB, depth, and skeleton data). The dataset is designed for user-independent evaluation to better reflect real-world conditions. Baseline models utilizing Convolutional Neural Networks (CNNs) for feature extraction and Long Short-Term Memory (LSTM) networks for temporal modeling were evaluated on AUTSL. The models achieved 96.11 % accuracy on the Montalbano dataset and up to 95.95 % on AUTSL with random splits, but only 62.02 % in the user-independent test, highlighting the challenges in real-world SLR applications. For morphologically rich languages like Turkish, traditional NLP methods require extensive pre-processing, including tokenization, lemmatization, and feature engineering, to address challenges such as data sparsity and high dimensionality. BERT's effectiveness in Turkish is investigated in [10], aiming to minimize these pre-processing burdens. The evaluation encompasses five Turkish NLP tasks—sentiment analysis, cyber-bullying identification, text classification, emotion recognition, and spam detection—and compares BERT's performance with that of baseline machine learning models. Results demonstrate that BERT outperforms traditional models, effectively handling Turkish text without the need for complex pre-processing. Tocoglu and Alpkocak, [11] evaluate machine learning models, including complement naive Bayes (CNB), random forest (RF), decision tree (C4.5/J48), and support vector machines (SVMs). It demonstrates that SVMs achieve the highest performance, with models trained on the validated dataset outperforming those trained on raw data. These findings emphasize the importance of dataset quality in emotion extraction tasks. Emotion extraction from text is a key task in natural language processing (NLP), requiring large, annotated datasets for supervised learning. However, for Turkish, there is a lack of comprehensive emotion-labeled datasets.

Four machine learning models for sentiment classification are developed and evaluated in [12], assessing their performance in both balanced and imbalanced scenarios. The best model achieves an F1-score of 0.81 for polarity classification and 0.39 for score classification. The dataset and fine-tuned models are openly available under the Creative Commons Attribution 4.0 International License on GitHub. Sentiment analysis for Kazakh has been hindered by a lack of large, annotated datasets. This study introduces KazSAnDRA, the first and largest publicly available dataset for Kazakh sentiment analysis, containing 180,064 reviews with ratings from 1 to 5. The dataset supports both polarity and score classification tasks.

Yeshpanov, et al. [13] introduce a Kazakh NER dataset annotated according to the IOB2 scheme, containing 112,702 sentences and 136,333 annotations across 25 entity classes. The dataset was annotated by two native Kazakh speakers under supervision to ensure consistency. State-of-the-art machine learning models for NER were developed, with the best model achieving an F1-score of 97.22 % on the test set. The dataset, annotation guidelines, and training codes are publicly available under the Creative Commons Attribution 4.0 License for further research. A rule-based punctuation analysis model [14] for Uzbek texts is proposed, addressing the lack of punctuation prediction tools for low-resource languages, with potential applications for other Turkic languages. Number of other computational approaches have been emergin in the Uzbek NLP research with various approaches from rule-mased, machine learning, AI, as well as statsitical models [15].

## 3 Data Description

All the data resides in one Microsoft Excel file (.xlsx) in the form of a simple table in a single sheet.

This dataset contains 9 columns in a plain text format with no extra elements like formulas or diagrams present. Below, we provide a description of these columns:

1. VERB NAME - This column contains the infinitive form of verbs.
2. The "PREVIOUS PART OF SPEECH" column indicates the word class from which the verb is derived. The following annotations are provided in the second column, indicating the preceding part of speech of the verb. *Sifat* - adjective, *ot* - noun, *fel* - verb, *taqlid* - onomatopoeia, *son* - number, *ravish* - adverb, *modal* - modal
3. The "DERIVATIONAL AFFIX" column contains the word-forming suffix of the verb.
4. The "COLLABORATIVE VOICE AFFIXES" column presents the affixes indicating the collaborative (reciprocal) voice of verbs.
5. The "INTENSIVE VOICE" column contains the affixes indicating the intensive (causative) voice of verbs.
6. The "PASSIVE VOICE" column contains the affixes indicating the passive voice of verbs.
7. The "REFLEXIVE VOICE" column contains the affixes indicating the reflexive voice of verbs.
8. The eighth and ninth columns will be INTENSIVE VOICE and COLLABORATIVE VOICE AFFIXES, respectively, because these affixes can also appear at the end. The total of 8502 verbs in this dataset are distributed as follows based on their derivation from previous parts of speech:

• Verb: 3461
• Noun: 2444
• Adjective: 1386
• Onomatopoeia: 1143
• Adverb: 35
• Numeral: 17

We can see that the **smallest number** of verbs, **17**, were derived from the **numeral** part of speech.

As mentioned above, each affix type included in this dataset modifies the verb in a specific way. For instance, the derivational affix *-la* in "tayinlamoq" (to assign) derives a verb from a noun (tayin – "sertain"). The collaborative affix -ish in "ko‘rishmoq" (to meet) means reciprocal action. The intensive affix -il in "yozilmoq" (to get listed in) adds passive voice. Another passive form example suffix is -in in "almashinmoq" (to get changed) shift the action focus.

Regarding the case of rare verb categories, the dataset includes examples of derivative types such as *"yigirmalanmoq"* (derived from the numeral *"yigirma"*, meaning "twenty") as well as *"gʻuvullamoq"* (derived from the onomatopoeic form *"gʻuvul"*), which represents the verb formation from numerals and sound-symbolic words, respectively.

Limitations. The major limitation point of the dataset is that it primarily covers the official written Uzbek verbs and does not consider or include regional dialect versions of verbs or colloquial verb forms, which may affect generalizability in broader linguistic applications.

## 4 Experimental Design, Materials and Methods

The list of verbs in the dataset was extracted from the five-volume Explanatory Dictionary of the Uzbek Language [1].

The Explanatory Dictionary of the Uzbek Language has been declared open-source and is available for use on the internet. It has been published under the CC BY 4.0 open license by the Uzbek Language Development Fund under the Cabinet of Ministers of the Republic of Uzbekistan.

Each verb extracted from the dictionary has been analyzed by specialists and segmented into affixes. The data extraction process was carried out half-manual and half-automated: verbs were at first identified and extracted using a custom script that parsed entries from the Explanatory Dictionary of the Uzbek Language. This automated step was followed by manual verification by experts in the linguistic field to make sure that the data is clean. Affixes were categorized based on manually-written grammatical rules, and ambiguous cases were resolved through agreement among multiple reviewers.

The verbs in the dataset are arranged in alphabetical order. The affixes in the verbs have been identified and re-checked by specialists. The preceding word class of each verb has also been determined, which is used in the morphological analysis of the verbs.

When filling in the information for each column in the dataset, the order of affixation in the verbs has been taken into account.

The presented verb lists are most suitable for the morphological analysis of Uzbek texts.

Verbs in the Uzbek language are responsible for conveying a significant amount of meaning beyond the simple lexical information. For example, by analyzing the affixes attached to a verb, we can determine not only the tense, aspect, and mood but also the voice (passive, reflexive, collaborative) and the intensity of the action (intensive). Thus, the ability to correctly identify and categorize these verb forms is critical for successful NLP tasks, such as **morphological analysis** and **syntactic parsing**. The dataset of Uzbek verbs in different forms provides the necessary foundation for building computational models capable of understanding and processing these linguistic features.

This structure is highly beneficial for researchers and developers in computational linguistics, as it:

• **Facilitates morphological analysis**: The dataset can be used to identify and classify different verb forms and affixes.
• **Supports NLP tools**: The organized structure makes it easier to input into language modeling tools, parsers, and machine learning algorithms designed for syntactic and semantic analysis.
• **Provides a foundation for further research**: It can serve as a benchmark for future studies on Uzbek verb morphology and its integration into NLP systems.

## Limitations

None.

## Ethics Statement

The data gathering process did not involve the use of human subjects. The only possibly problematic ethical aspect comes from the possible privacy concerns of the gathered corpus and consequently the extracted word lists. All the gathered texts are from publicly available web pages in Uzbek language.

## Credit Author Statement

**Maksud Sharipov:** Conceptualization, Methodology, Formal analysis, Resources, Supervision, Writing - Original Draft, Writing – Review & Editing, Project administration. **Jernej Vičič:** Conceptualization, Investigation, Writing – Review & Editing, Data Curation.

## Data Availability

Mendeley DataDataset of Uzbek verbs with formation and suffixes (Reference data).
